# Frantz's Tumor in Focus: The Tale of a 34-Year-Old Yemeni Female Patient

**DOI:** 10.7759/cureus.45258

**Published:** 2023-09-14

**Authors:** Saleh A Ba-shammakh, Bourhan Alrayes, Uwise Awaisheh, HISHAM HAMAD, Mohammad S AL-Qannas, Hamza M Abu-obead

**Affiliations:** 1 Department of General Surgery, The Islamic Hospital, Amman, JOR

**Keywords:** pancreatic neoplasm, beta-catenin, portal vein reconstruction, whipple procedure, frantz's tumor, pancreas, solid pseudopapillary tumor

## Abstract

A solid pseudopapillary tumor (SPT) of the pancreas, which mainly occurs in young women, is an uncommon pancreatic tumor that often presents diagnostic and therapeutic dilemmas. This case study discusses the symptoms and treatment approach for a 34-year-old woman from Yemen diagnosed with SPT. The patient was diagnosed through abdominal and pelvis CT scan, followed by ultrasound-guided biopsy confirming the presence of SPPT. Management through the Whipple procedure and portal vein reconstruction proved successful, with no recurrence or metastasis noted in a year-long follow-up. The importance of comprehensive understanding and surgical expertise in handling SPT is emphasized.

## Introduction

A solid pseudopapillary tumor (SPT) of the pancreas, sometimes referred to as pancreatic solid pseudopapillary neoplasm (SPN) or Frantz's tumor, represents a unique pancreatic tumor type, making up roughly 3% of all exocrine pancreatic tumors [1,2]. The data indicates that the neoplasm primarily affects individuals aged between 20 to 40 years [2-3]. Within this age group, over 90% of the identified cases are found in young women aged between 20 and 30. Recent trends suggest an upswing in diagnosed cases over the last two decades, potentially due to advancements in diagnostic techniques [2-5]. Often, these patients don't show symptoms, but some might experience a slowly growing abdominal mass, or in rare cases, jaundice [6]. While the exact pathophysiology of SPTs remains shrouded in uncertainty, various hypotheses have been put forth. Some researchers propose its origin from primordial multipotent cells, while others speculate an extra-pancreatic genesis [7]. Regarding its prevalence across different ethnic groups, it appears that African American and Asian women are at a higher risk, with an observed female-to-male ratio of 10:1 [6]. Numerous studies have noted the tumor often manifests in the body and tail regions of the pancreas [8-9]. Despite the challenges it poses, the primary therapeutic recommendation remains surgical resection with clear margins [8]. Prognostically, patients with SPN or SPT generally fare well, enjoying an extended survival span that often surpasses 50 months. However, rare instances of complications like peritoneal carcinomatosis have been reported, potentially stemming from capsule rupture during surgical procedures or other external trauma [8-9].

## Case presentation

A 34-year-old Yemeni woman had been grappling with abdominal pain for five months, which initially led to her admission to the hospital. This pain was intermittent, non-radiating, gradual onset, had a severity of 5/10, and did not correlate with her eating or defecation patterns. Interestingly, it could be partially eased with analgesia. The patient didn't report any recent weight loss, night sweats, or changes in bowel habits. With no record of previous similar attacks, a family history of the same, chronic illness, history of surgery, or allergies, she was an otherwise healthy individual. Regarding her social history, she was a non-smoker and teetotaller, with no family history of malignancy.

Upon admission, she was fully conscious, alert, and oriented. Her vital signs were stable, with a body temperature of 37.3°C, heart rate of 72 bpm, respiratory rate of 22 breaths/min, blood pressure reading 100/70 mmHg, and oxygen saturation at 98%. She showed no signs of jaundice, or pallor, and no palpable lymph nodes were noted. Her respiratory system was unremarkable. Regarding abdominal examination, a palpable firm-mild tender mass in the right upper quadrant (RUQ) was noted. The skin over the mass displayed no changes. 

**Figure 1:**
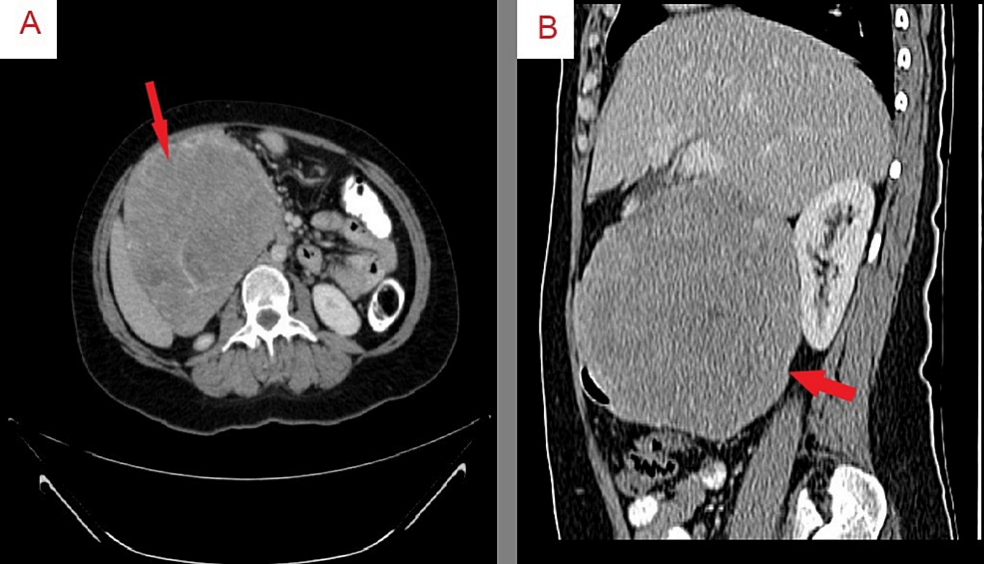
CT scan of the abdomen and pelvis (A) Venous phase axial CT scan of the abdomen illustrating a large, heterogeneous, encapsulated solid mass with necrosis in the porta hepatis area. The mass measures 13 x 12 x 16 cm in TS, AP, and CC dimensions respectively, and is seen causing a mass effect on the surrounding structures. (B) Venous phase sagittal CT scan depicting the extent and orientation of the mass relative to surrounding anatomical structures.

Our initial laboratory tests (Table 1) yielded normal renal function, electrolytes, and liver function, with a slightly raised C-reactive protein level of 23 mg/L, implying a presence of inflammation. Her urinalysis was unremarkable. An abdomen and pelvis CT scan with IV, oral, and rectal contrast was conducted, exposing a large, heterogeneous, encapsulated solid mass with necrosis in the porta hepatis area. This mass, which measured 13 x 12 x 16 cm in the TS, AP, and CC dimensions respectively, was located near the right hepatic artery and portal vein, causing a mass effect on the surrounding structures (Figure 1). There was no evidence of lymphadenopathy, free fluid, or pneumoperitoneum.

**Table 1:** laboratory tests PH: potential hydrogen; WBCS/HPF: white blood cells per high power field; RBCs/HPF: red blood cells per high power field serum chemistry; mg/dL: milligrams per deciliter; mmol/L: millimoles per liter; U/L: units per liter; LDH: lactate dehydrogenase

Test	Result	Reference Range
Urine Routine & Microscopy		
Specific Gravity	1.025	1.003 - 1.030
Nitrite	Negative	
PH	5	4.6 - 8
Protein	Negative	
Glucose	Normal	
Ketones	Negative	
Urobilinogen	Normal	
Bilirubin	Negative	
Blood	Trace	
WBCs/HPF	0-2	<5/HPF
RBCs/HPF	0-1	0-2/HPF
Epithelial cells	Moderate	
Casts	Not seen	
Crystals	Amorphous Urate	
Others	Not seen	
Serum Chemistry		
Creatinine	0.59 mg/dL	Female & Child: 0.5-1.0 mg/dL; Male: 0.7-1.2 mg/dL
Sodium	136 mmol/L	136-145 mmol/L
Potassium	3.81 mmol/L	3.5-5.3 mmol/L
Chloride	102.3 mmol/L	98-107 mmol/L
Calcium	9.34 mg/dL	Adult: 8.6-10.2 mg/dL; Child: 9.0-11.0 mg/dL
Phosphorus	2.64 mg/dL	Adult: 2.5-4.5 mg/dL; Infant: 3.5-7 mg/dL
Uric Acid	4.6 mg/dL	Female: 2.4-5.7 mg/dL; Male: 3.4-7.0 mg/dL
LDH Total	177 U/L	Adult Female: 135-214 U/L; Adult Male: 135-225 U/L; 2-15 Years: 120-300 U/L
C-reactive Protein	23 mg/L	Adult: <5.0 mg/dL
Infection Control		
HBs Ag	Negative	
Hepatitis C AB	Negative	
Chemistry		
Total Protein	7.48 g/dL	Adult: 6.4-8.3 g/dL; Children: 5.6-8.0 g/dL
Albumin	4.69 g/dL	3.5-5.2 g/dL
Total Bilirubin	0.74 mg/dL	Male: Up to 1.2 mg/dL; Female & Child: Up to 1.0 mg/dL
Alkaline Phosphatase Total	92 U/L	44-147 IU/L

**Figure 2:**
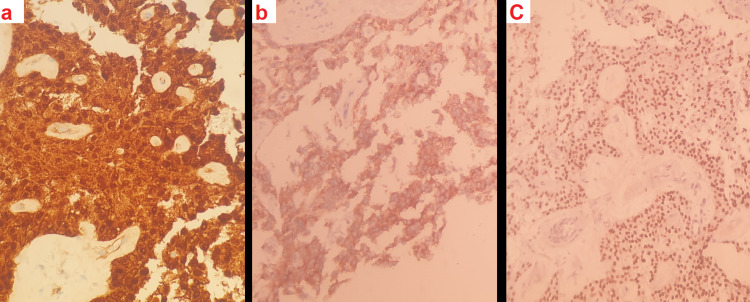
Histopathological slide (a)This figure showcases the proliferation of monomorphic cells with ovoid nuclei arranged in small solid sheets. Positive immunohistochemistry stains for B-catenin, CD10 (b), and progesterone receptors (c) confirm the diagnosis of a solid pseudopapillary tumor (SPPT) of the pancreas.

A subsequent ultrasound-guided biopsy of the mass showcased a proliferation of monomorphic cells with ovoid nuclei arranged in small solid sheets. The immunohistochemistry results were positive for B-catenin, CD10, and progesterone receptors, which led us to a diagnosis of a solid pseudopapillary tumor (SPPT) of the pancreas (Figure 2).

**Figure 3:**
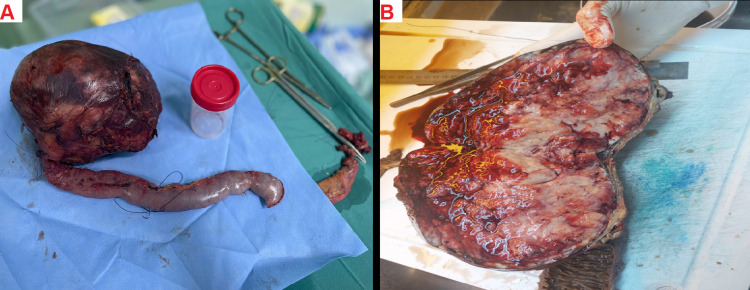
Intraoperative figures (A) The image shows A 2 kg, 25x23x10 cm, well-demarcated, partially encapsulated mass with a solid, soft-cut surface with areas of hemorrhage. The mass is adherent to the 5x4x1.5 cm part of pancreatic tissue, as well as to 12 cm of attached duodenum and a 34 cm segment of the jejunum. (B) The image shows the cross-sectional cut of the mass and its encapsulation and area of hemorrhage as well as the necrosis inside the parenchyma of the tumor alongside a cross-sectional cut of the jejunum. There are infiltrative areas into adjacent pancreatic tissue, as well as infiltration into the portal vein wall but not reaching the lumen.

In light of the biopsy findings and her persistent symptoms, a very challenging Whipple procedure with portal vein excision and reconstruction was performed on October 22, 2022. A large vascular mass, originating from the head of the pancreas, was discovered without evidence of metastatic disease (Figure 3).

**Figure 4:**
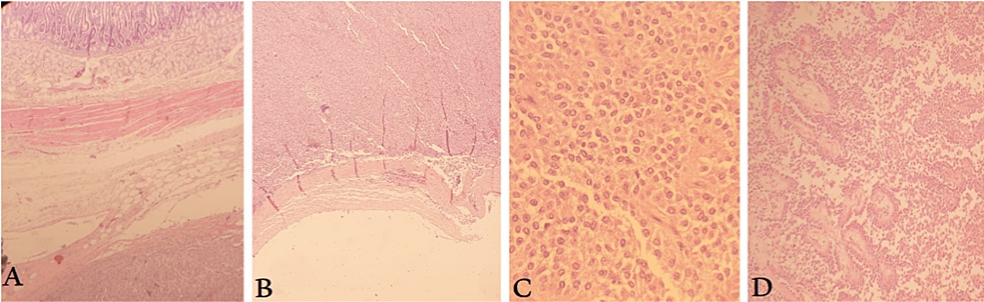
Microscopic section of the tumor (A-B) Low power field (10X) H&E highlighting encapsulation of the tumor separating it from the adjacent structure (jejunum). (C-D) High power field (40X) H&E depicts monomorphic cells with scattered foamy appearance and ovoid nuclei. The mitotic activity is low. Notable are nuclear pseudoinclusions, eosinophilic and clear cytoplasm arranged in small solid sheets. Additionally, there are trabecular and acinic-like structures with interspersed areas of thick hyaline bundles. These features align with a pancreatic tumor having characteristics of a solid pseudopapillary tumor.

Transfusion of four packed RBC cell units as well as two free frozen plasma and two platelets units in total was given intraoperatively. Post-operative analysis of the resected mass confirmed our preoperative diagnosis of SPPT. The tumor had infiltrated the adjacent pancreatic tissue and the portal vein wall, necessitating their urgent excision. However, there was no infiltration into the duodenal wall or the portal vein lumen (Figure 4). The resected lymph nodes didn't show any signs of tumor involvement.

Following the surgery, she was moved to the ICU for five days, where she was kept nil per oral (NPO) and placed on IV fluids. Her pain was managed with a patient-controlled analgesia (PCA) pump. On the third postoperative day, the patient exhibited severe chest pain accompanied by tachycardia, symptoms often associated with significant anxiety. Given the gravity of her symptoms, immediate steps were undertaken to rule out potential cardiac complications and pulmonary embolism (PE) through appropriate diagnostic procedures. These investigations confirmed that these were not the underlying causes of her distress. Consequently, a daily regimen of bisoprolol 2.5 mg was initiated, which resulted in a notable improvement in her condition. Her diet was gradually reintroduced with clear fluids, which she tolerated well. She had follow-up appointments at three- and six-month intervals which revealed no other symptoms. There was no recurrence of symptoms or signs of recurrence, metastasis, or any complications.

## Discussion

Solid pseudopapillary neoplasm (SPN) of the pancreas, often referred to as Frantz's tumor, is a fascinating diagnostic entity. It was first described by Virginia Frantz in 1959 [10]. Even though its initial prevalence was unclear, questions about its malignant nature persist, even decades after its original recognition [1].

SPN was added to the WHO classification in 1996 [11]. The tumor's nomenclature can be traced back to Dr. Virginia Kneeland Frantz, who first documented it as a papillary-cystic tumor in the pancreas [1]. Undoubtedly, this condition is rare, and its low potential for malignancy usually indicates a positive prognosis [1]. It predominantly affects females, showing a striking female-to-male ratio of 10:1 [2]. Notably, the frequency of reported cases has significantly surged in the recent two decades, not due to an increased incidence but due to advancements in imaging modalities and increased awareness [12].

The symptoms range from nonspecific abdominal discomfort and pain to compression symptoms due to the expansive nature of the mass [13]. Physical examinations, while mostly inconclusive, can occasionally reveal palpable large masses. Imaging, especially CT scans, plays a pivotal role in diagnosing these neoplasms. Most tumors are incidental findings, with patients largely being asymptomatic [13]. Tumors have the potential to develop into sizable growths, sometimes growing larger than 15 cm [2]. The favored technique for diagnosing these types of pancreatic tumors is through endoscopic ultrasound-directed fine needle aspiration [14].

The hallmark histological features of this condition are solid tissue sections that create pseudo-papillae, mixed with cystic regions showing signs of hemorrhage [15]. It's these specific characteristics that are crucial for identifying the tissue [13,16]. When examining it on a cellular basis, the cells of SPN frequently show the presence of beta-catenin, CD10, and CD56. However, they typically lack pancreatic enzymes and chromogranin [13,17]. Importantly, every SPN is associated with mutations in the beta-catenin pathway [18].

Surgical resection remains the cornerstone of SPN management. Most patients undergoing surgical excision find it curative [19-20]. However, while metastasis is rare, it's not a contraindication to surgical removal. The most common metastasis site is the liver [16]. Surgical methods employed depend on the tumor location; for instance, tumors in the pancreas head often warrant a duodenopancreatectomy [21]. It is rare that these tumors push into the portal vein; this mandates the urgent excision and reconstruction of the tumor, as in our case. 

The prognosis for SPN is generally positive, with a few cases recording recurrence post-excision [22-23]. Factors such as perineural invasion, angioinvasion, larger tumor size, and high mitotic rate can increase the recurrence risk of SPNs [24]. Although recurrences are relatively rare, the liver and peritoneum are the most frequent metastasis sites [25]. Recent literature has shown that with a multidisciplinary approach, five-year survival rates can exceed 50% [25]. Despite its usually indolent nature, long-term patient follow-up post-surgical resection is essential, especially considering cases like the one discussed in Vassos et al., where an incidental pancreatic tumor diagnosis occurred post-abdominal trauma [26-27].

SPN, with its challenging diagnosis, has seen an increase in reporting over the last two decades due to advancements in imaging techniques. Despite its generally favorable prognosis, a comprehensive understanding of its pathology, combined with surgical expertise, is essential to manage these neoplasms effectively. The need for further exploration into its histopathological features and potential markers cannot be understated, as they may offer insights into recurrence potential and overall prognosis [26-27].

## Conclusions

Solid pseudopapillary neoplasm (SPN) of the pancreas remains a diagnostic and therapeutic challenge, largely due to its rarity. This case underscores the significance of modern imaging techniques in diagnosing such neoplasms. Surgical resection stands as the most effective therapeutic strategy. Although SPN patients generally have a positive prognosis, long-term postoperative monitoring is crucial. The increasing reports of SPN in recent years highlight the importance of continued research to better understand its pathology, ensuring effective management and better patient outcomes.
